# Electrochemical Sensing Fabricated with Ta_2_O_5_ Nanoparticle-Electrochemically Reduced Graphene Oxide Nanocomposite for the Detection of Oxytetracycline

**DOI:** 10.3390/biom10010110

**Published:** 2020-01-08

**Authors:** Felista Magesa, Yiyong Wu, Shuai Dong, Yaling Tian, Guangli Li, John Mary Vianney, Joram Buza, Jun Liu, Quanguo He

**Affiliations:** 1 School of Life Sciences and Chemistry, Hunan University of Technology, Zhuzhou 412007, China; magesaf@nm-aist.ac.tz (F.M.); wyy5082010@163.com (Y.W.); 15674180029@163.com (S.D.); tianyaling0212@163.com (Y.T.); guangli010@hut.edu.cn (G.L.); 2 School of Life Sciences and Bioengineering, Nelson Mandela African Institution of Science and Technology, P.O. Box 447, Arusha, Tanzania; john-mary.vianney@nm-aist.ac.tz (J.M.V.); joram.buza@nm-aist.ac.tz (J.B.)

**Keywords:** Ta_2_O_5_-ErGO composition, oxytetracycline, modified electrode, electrochemical reduced graphene oxide, voltammetric determination

## Abstract

A novel tantalum pentoxide nanoparticle-electrochemically reduced graphene oxide nanocomposite-modified glassy carbon electrode (Ta_2_O_5_-ErGO/GCE) was developed for the detection of oxytetracycline in milk. The composition, structure and morphology of GO, Ta_2_O_5_, and Ta_2_O_5_-ErGO were characterized by X-ray diffraction (XRD) and scanning electron microscopy (SEM). Oxytetracycline electrochemical behavior on the bare GCE, GO/GCE, ErGO/GCE, and Ta_2_O_5_-ErGO/GCE was studied by cyclic voltammetry. The voltammetric conditions (including scan rate, pH, deposition potential, and deposition time) were systematically optimized. With the spacious electrochemical active area, the Ta_2_O_5_-ErGO/GCE showed a great magnification of the oxidation signal of oxytetracycline, while that of the other electrodes (GCE, GO/GCE, ErGO/GCE) could not reach the same level. Under the optimum conditions, the currents were proportional to the oxytetracycline concentration in the range from 0.2 to 10 μM, and a low detection limit of 0.095 μM (S/N = 3) was detectable. Moreover, the proposed Ta_2_O_5_-ErGO/GCE performed practically with satisfactory results. The preparation of Ta_2_O_5_-ErGO/GCE in the current work provides a minor outlook of detecting trace oxytetracycline in milk.

## 1. Introduction

Antimicrobial residues in food products of animal origin have become a great global safety concern. Oxytetracycline (OTC) is one of the antibiotics in the tetracycline group. It is commonly used to treat and prevent bacterial infections in livestock, as well as growth promotion. However, the non-prudent use of OTC in animals leads to accumulation of the residues in animal products such as milk. Small doses of antibiotic exposure to humans through the food chain poses health threats including hypersensitivity [1,2], antibacterial drug resistance [3], and toxic effects [4]. In protecting the public from exposure to residues, different regulatory organizations (Food and Agriculture Organization, European Union, and Food and Drug Authority) have set a maximum residue limit (MRL) of 0.1 mg/L in milk [4,5,6]. Thus, the detection of these residues in food products is the first critical step of control. Different analytical methods have been employed in the determination of OTC in milk. The methods that are conventional so far include liquid chromatography-tandem mass spectrometry, high-performance liquid chromatography (HPLC) [5], enzyme-linked immunosorbent assays (ELISA) [6], and surface-enhanced Raman scattering (SERS) [7]. These methods are sensitive but laborious, time-consuming, have extensive equipment requirements and cannot be applied easily in the field. Hence, novel, portable, rapid, sensitive, specific, and environmentally friendly analytical tools are highly demanded. Recently, there has been a rise in the development and use of electrochemical analysis techniques in the detection of OTC. A few such as photoelectrochemical (PEC) [8,9] and fluorescence [10,11] have shown great promise for use in sensing applications.

Nanoparticles have gained tremendous interest in the fields of sensing, catalysis, energy storage, and biomedical [12] applications, especially transition metal oxide nanoparticles. Scientists and researchers are highly applying these nanoparticles such as Cu_2_O [13,14,15,16,17,18], ZnO [19,20], MnO_2_ [21,22,23,24,25,26], Fe_2_O_3_ [27,28], TiO_2_ [29,30], and many others [31,32,33], since they have tunable band gaps and, hence, wide application, good thermal and chemical stability, good conductivity, and excellent photoelectric performance. Among the many semiconductors, and the few mentioned above, Ta_2_O_5_ is one of the very significant transition metal oxides and has recently attracted attention due to its unique characteristics, which include excellent photoelectric ability, high dielectric coefficient, high refractive index, good chemical stability as well as biological and electro-catalytic activity. Therefore, Ta_2_O_5_ metal–oxide semiconductor nanoparticle applications have been widely studied. It has been reported by many studies that the material is showing wide application in the photo-catalysis [29,34,35,36] and biomedical applications [20]. In addition, there are promising results in the use of Ta_2_O_5_ NPs in the electrochemical sensing of toxic chemical substances in foods and human samples [37,38]. However, in sensing chemical contaminants such as food additives and antibiotic residues in food and food products, more effort is needed in exploring the potential of the material as other transition metal oxide nanoparticles have shown good results in the mentioned area.

Graphene (GR) is a novel material, consisting of a single-atom-thick planar sheet with sp^2^ bonded carbon structure. The material has been reported to be ideal in application in a wide range of fields such as sensing, catalysis, electronic devices, and energy storage [39]. Graphene as an emerging material is rapidly rising because of its outstanding characteristics such as high mechanical strength [40,41], large surface area, extraordinary electron transfer capacity, and good thermal and electric conductivity [42]. In addition, graphene derivatives such as graphene oxide (GO) and reduced grapheneoxide (rGO) have also shown great promise in electrochemical sensing applications. GO is a 2D material consisting of functional groups that contain oxygen. The functional groups containing oxygen that have been mostly reported include epoxy and hydroxyl groups, which are located on the basal plane of the sheet. It has also been identified to contain few amounts oflactone, quinone, carboxy, carbonyl, and phenol at the edges of the sheet. These functional groups have a significant effect on GO electrochemical properties and activity [42]. The latter makes GO a good material for modification such as reduction to form rGO, and immobilization with other electro-active materials such transition metaloxides in order to form an excellent conductive material for electrochemical sensing platforms. Zhou et al. and Sun et al. [29,37] have reported the successful application of the Ta_2_O_5_/GO nanocomposite synergistic effects inelectrochemical sensing and photocatalysis, respectively. This gives insight that there is a potential application in the combined effect of the Ta_2_O_5_/GO nanocomposites.

**Scheme 1:**
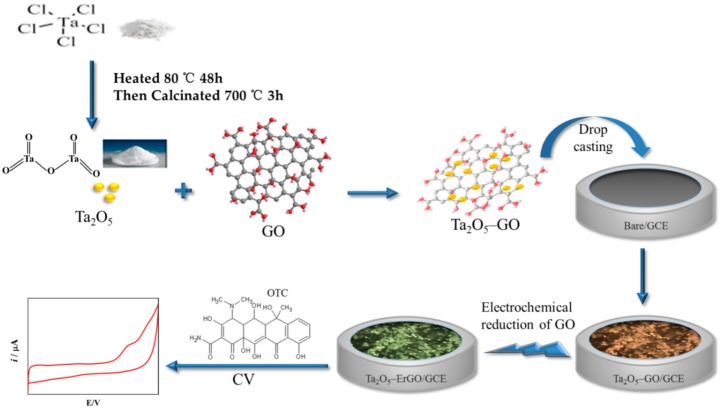
The preparation of a tantalum pentoxide nanoparticle-electrochemically reduced graphene oxide nanocomposite-modified glassy carbon electrode (Ta_2_O_5_-rGO/GCE) for the determination of tryptophan.

The current work investigated the use of a glassy carbon electrode (GCE) fabricated with Ta_2_O_5_-electrochemically (E)rGO nanocomposites for the determination of OTC. Ta_2_O_5_ NPs were synthesized through the hydrothermal-calcination method. Ta_2_O_5_ was firmly immobilized on the GO through the nucleation sites provided by the oxygen functional groups to form Ta_2_O_5_-GO nanocomposites. Then, through electrochemical reduction, Ta_2_O_5_-ErGO nanocomposites were formed (Scheme 1). The resulting material indicated good electrochemical activity on the determination of OTC. Eventually, it was used in detecting OTC in milk.

## 2. Experimental

### 2.1. Chemicals and Solutions

Graphite powder, tantalum chloride (TaCl_5_), potassium permanganate (KMnO_4_), diethanol amine (DEOA), hydrogen peroxide (H_2_O_2_), sulfuric acid (H_2_SO_4_), sodium hydroxide (NaOH), trichloro acetic acid, potassium ferricyanide (K_3_Fe(CN)_6_), α-aluminum oxide (α-Al_2_O_3_, particle size: 1.0, 0.3, and 0.05 µm), and ethyl alcoholwere purchased from Sinopharm Chemical Reagent Company (Shanghai, China). Disodium phosphate dodecahydrate (Na_2_HPO_4_·12H_2_O), sodium dihydrogen phosphate (NaH_2_PO_4_), and oxytetracycline (OTC) were provided by Aladdin Bio-Chem Technology Co., Ltd. (Shanghai, China). The milk, which needed to be pretreated, was taken from the supermarket (Zhuzhou, Hunan, China).

### 2.2. Equipment and Apparatus

Voltammograms were obtained through a cyclic voltammetry (CV) electrochemical technique using a CHI 660E electrochemical workstation from the company of Shanghai Chenhua Instruments, China. The experiment conducted involved the use of GCE as the working electrode, which was fabricated with a Ta_2_O_5_ NPs-ErGO, platinum electrode and saturated calomel electrode as the counter and reference electrodes respectively. The pH analysis was conducted ona pH-3c exact digitalpH meter (Leichi Instrument Factory, Shanghai, China). Scanning electron microscopy was performed and images were obtained at an acceleration voltage of 2.0 kV by a scanning electron microscope (EVO10, ZEISS, Jena, Germany). The crystalline structure analysis of Ta_2_O_5-_was operated with Cu Kα radiation (0.1542 nm) ona powder X-ray diffractometer (PANalytical, Amsterdam, Holland).

### 2.3. Ta_2_O_5_ Nanoparticle Preparation

The hydrothermal method was applied in Ta_2_O_5_ preparation [34]. Then, 0.01 M NaOH 100 mL) was poured rapidly into 300 mL of 0.05 M TaCl_5_ containing 0.1ml of diethanolamine, which was added as a stabilizer. The solution was stirred for 1 h at room temperature and transferred into a hydrothermal autoclave, and then heated at 80 °C for 48 h for crystallization to occur. Upon cooling to room temperature, the powder was washed using distilled water until no precipitate remained between the filtrate and AgNO_3_. To remove impurities, the powder was washed with ethanol three times and dried at room temperature using a vacuum drier. To obtain Ta_2_O_5_ nanoparticles, the dried powder was calcinated at 700 °C for 3 h in a muffle furnace.

### 2.4. Preparation of Ta_2_O_5_-GO

Commercial GO was used in preparing the dispersion. 20 mg of GO powder was mixed with water (20 mL) to obtain a solution of GO with the concentration of 1 mg/mL. Then, 1 mg Ta_2_O_5_ nanoparticles was mixed in 10 mL of the prepared GO solution. Subsequently, the mixed solution was ultrasonicated for 2 h to form a Ta_2_O_5_-GO dispersion.

### 2.5. Electrode Fabrication

A glassy carbon electrode (GCE) surface (3 mm in diameter) was polished with different fine sizes (1.0, 0.3, and 0.05 µm) of α-Al_2_O_3_ powder and then ultrasonicated in distilled water, absolute ethanol, and then distilled water for one minute in each and left to dry under an infrared lamp. The Ta_2_O_5_-GO dispersion (5μL) was drop-casted on the clean GCE surface and left to dry on air. The Ta_2_O_5_-ErGO/GCE was prepared by dipping the Ta_2_O_5_-GO/GCE in 0.1 M phosphate buffer solution (PBS, pH 6.5), and the reduction process was run at a constant potential of −1.2 V for 120 s. For the purpose of comparing the electrodes, a similar method was used to prepare other fabricated electrodes such as a GO/GCE, ErGO/GCE, and Ta_2_O_5_-GO/GCE.

### 2.6. Real Sample Pretreatment

Whole milk samples were bought from local supermarkets. For the detection of OTC, the milk samples were pretreated as follows: Weighing 3 g of trichloroacetic acid, and then dissolving it in 100 mL of PBS pH 6.5 solution. Next, 15 mL of milk was put in a 50 mL centrifuge tube, and the addition of 15 mL of the dissolved trichloroacetic acid was then followed by gently shaking. After that, centrifugation was done at 4000 rpm for 30 min. In order to obtain a very clear solution, the supernatant was filtered using the 0.22 µm pore filter. For practical testing of the developed sensor, milk was spiked with different concentrations of OTC.

### 2.7. Electrochemical Measurements

All the processes for material synthesis and electrochemical measurements are shown in Scheme 1. All electrochemical experiments were carried out by cyclic voltammetry (CV) with the three electrode systems (bare or modified GCE serving as a working electrode, platinum wire electrode serving as a counter electrode, and saturated calomel electrode (SCE) serving as a reference electrode). Oxytetracycline of 10 μM was prepared in 1.0 M PBS (pH 6.5). With the deposition time (90 s) and potential (0 V), the cyclic voltammetry was recorded on the electrodes. The calibration curve was established by plotting the relationship between the measured current signal and analyte concentration. The amount of oxytetracycline in milk sample solutions was obtained by the standard addition method.

## 3. Results and Discussion

### 3.1. Materials Characterization

**Figure 1:**
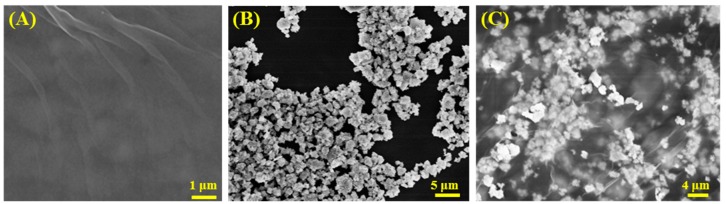
Electron microscopy images of the (**A**) pure graphene oxide (GO), (**B**) pure Ta_2_O_5_ nanoparticles, and (**C**) Ta_2_O_5_-GO composite nanoparticles.

The morphologies of the different synthesized materials were first explored by SEM. As Figure 1A shows, profuse plication and a crumple-like surface structure were displayed, demonstrating the successful preparation of GO. Figure 1B shows the typical image of Ta_2_O_5_ nanoparticles. Each homogeneous particle is clearly observed. The estimated size is 750 nm, and the large particles are formed by the small particles with the aggregation. Though these small particles are smaller than 100 nm, the true size could not be measured with the low resolution of the scanning electron microscope images. The morphology of Ta_2_O_5_-GO is clearly shown in Figure 1C. The majority of nanoparticles are uniformly dispersed on the surface of GO, illustrating that the Ta_2_O_5_ composited with GO nanosheets shows the excellent dispersity in contrast to the pure Ta_2_O_5_. Compared to the pure GO, Ta_2_O_5_-GO with an enormous specific surface area provides extra active sites that would absorb sufficient analytes.

**Figure 2:**
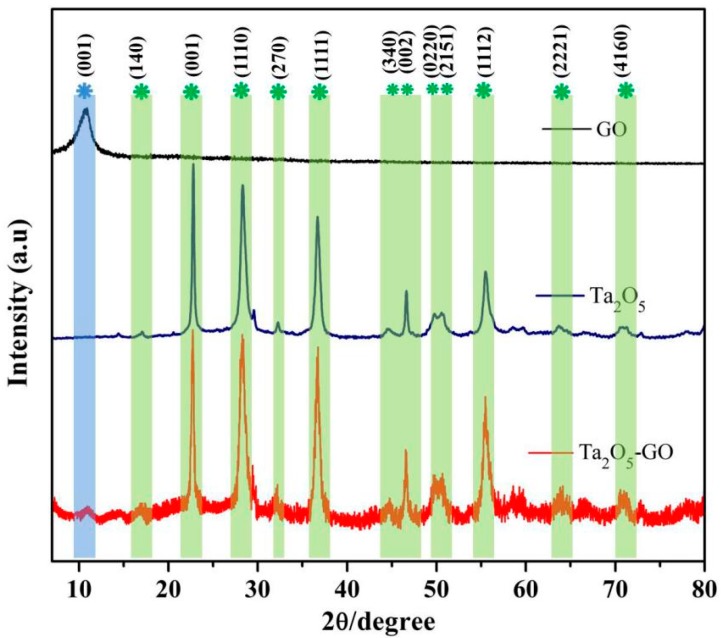
Patterns of pure Ta_2_O_5_, Ta_2_O_5_-GO composites, and graphene oxide (GO) nanosheets.

The crystalline structure of the GO, pure Ta_2_O_5_ nanoparticles, and Ta_2_O_5_-GO composite nanoparticles were further investigated by X-ray diffraction (XRD). As Figure 2 presents, the (001) plane corresponding to the blue column belongs to the typical XRD pattern of pure GO. Moreover, obvious diffraction peaks of Ta_2_O_5_ are observed at 2θ = 16.8°, 22.9°, 28.3°, 32.8°, 36.7°, 44.7°, 46.7°, 49.7°, 50.7°, 55.5°, 63.6°, and 71.4°, corresponding to the green columns, which are related to the (140), (001), (1110), (270), (1111), (340), (002), (0220), (2151), (1112), (2221), and (4160) reflections, respectively. The result agrees with the standard card (Joint Committee Powder Diffraction Standards, JCPDS, No. 25-0922) of Ta_2_O_5_ [37], demonstrating that the synthetic Ta_2_O_5_ nanoparticles were successfully prepared. Most importantly, not only the plane of GO, but also the those of Ta_2_O_5_ are observed in the XRD pattern of pure GO and Ta_2_O_5_-GO composites, indicating the successful synthesis of Ta_2_O_5_-GO composites.

### 3.2. Electrochemical Characterization of the Modified Electrodes

**Figure 3:**
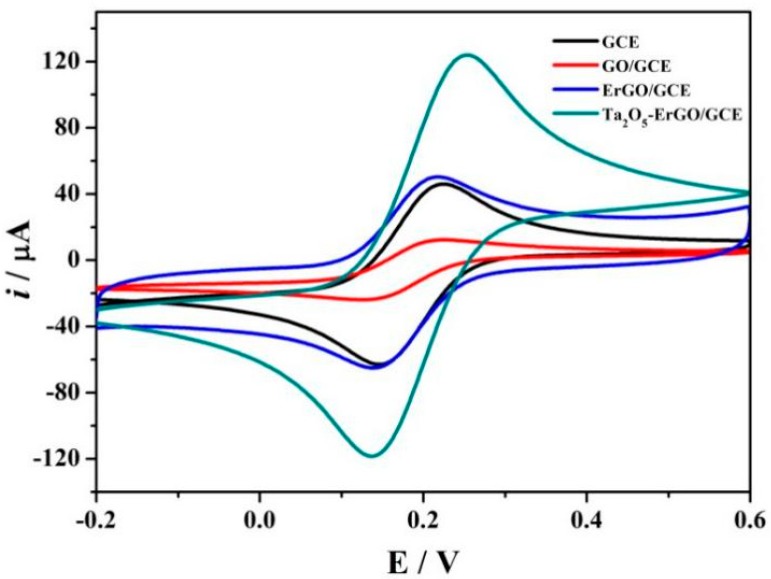
Cyclic voltammetry (CV) obtained on bare GCE, GO/GCE, ErGO/GCE, and Ta_2_O_5_-ErGO/GCE in a 5.0 mM K_3_Fe(CN)_6_ solution and 0.1 M KCL at the scan of 0.1 V s^−1^.

The cyclic voltammograms of bare GCE, GO/GCE, ErGO/GCE, and Ta_2_O_5_-ErGO/GCE were obtained in K_3_[Fe(CN)_6_] containing 0.1 M KCl, as indicated in Figure 3. As *i*_pa_/*i*_pc_ ≈ 1, the redox peaks appearing in each electrode tested were a pair of quasi-reversible ones. At the bare GCE, the potential separation (∆*E*_p_) of the redox peaks was 0.079 V with the weak current of 60.52 (*i*_pa_) μA and 63.87 (*i*_pc_) μA, while with the presence of the poor conductive material GO, the redox peak currents of GO/GCE decreased precipitously with *i*_pa_ = 23.96 μA and *i*_p__c_ = 24.18 μA. Moreover, after electrochemical reduction of GO to ErGO, the current on ErGO/GCE was higher than that of GO/GCE, implying a better conductivity of ErGO than that of GO. The intensity of the anodic and cathodic peak current of ErGO was 51.13 and 55.71 µA, respectively. Above all, the largest redox peak current (*i*_pa_ = 135.7 μA, *i*_pc_ = 134.5 μA) occurred to Ta_2_O_5_-ErGO/GCE, which approximately increased by twofold. The result shows that Ta_2_O_5_-ErGO/GCE dramatically improved electrochemical performances owing to the large specific surface area. For further investigation, the effective electroactive areas of various electrodes could be figured out by the Randles–Sevcik equation [21,22]:

*i*_pc_ = 2.691 × 10^5^ n^3/2^ D^1/2^*v*^1/2^ AC (1)

where the reduction peak current of K_3_[Fe(CN)_6_] is indicated as *i*_pc_; the number of electrons transferred during the redox process is indicated as n; the electrochemical active area (cm^2^) is indicated as A; the diffusion coefficient of K_3_[Fe(CN)_6_] is indicated as D (=7.6 × 10^−6^ cm^2^·s^−1^); the K_3_[Fe(CN)_6_] concentration in (mol·cm^−3^) is indicated as C; and the scanning rate (V·s^−1^) is indicated as *v*. Thus, the electrochemical active areas of bare GCE, GO/GCE, ErGO/GCE, and Ta_2_O_5_-ErGO/GCE can be worked out as 0.0545, 0.0206, 0.0475, and 0.1147 cm^2^, respectively. The Ta_2_O_5_-ErGO/GCE electrochemical active area is almost twofold that of bare GCE, elucidating that the Ta_2_O_5_-ErGO composite modification enables the effective electroactive surface area to expand, promoting the adsorption of oxytetracycline on the electrode surface, and thus facilitating the redox process of oxytetracycline.

### 3.3. Electrochemical Behavior of Oxytetracyclineon Various Electrodes

**Figure 4:**
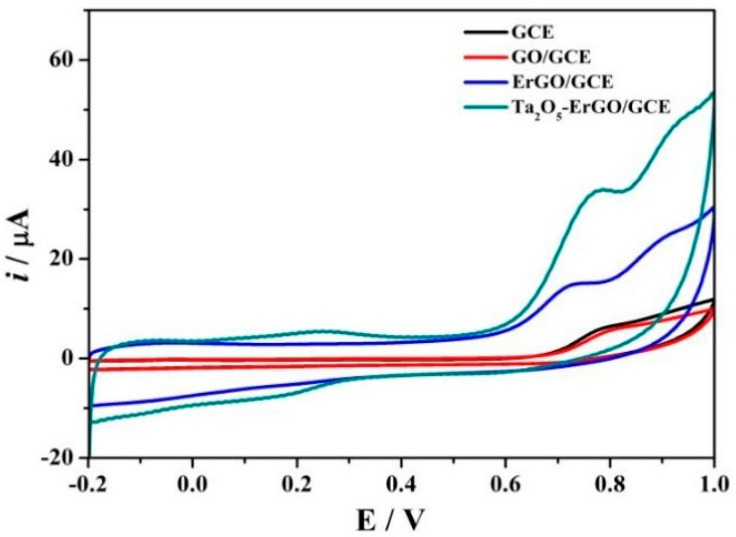
Oxytetracycline (10 μM) on bare GCE, GO/GCE, ErGO/GCE, and Ta_2_O_5_-ErGO/GCE (cyclic voltammetry, electrolyte: pH = 6.5, phosphate buffer solution (PBS), scan rate: 0.1 V/s).

Figure 4 displays the CV responses of 10 μM OTC in 0.1 M PBS obtained at various electrodes in the potential range from −0.2 to 1.0 V. Obviously, as shown in Figure 4, there are no cathodic peaks at all electrodes, while a redox peak is presented, illustrating that the oxidation of OTC is an irreversible process. Relatively, there is a weak anodic peak observed clearly at the bare GCE, which is similar to that of the GO/GCE. Most importantly, after the electrochemical reduction, the signal of the peak current of ErGO is magnified, but it is still less than that of Ta_2_O_5_-ErGO. This study shows that Ta_2_O_5_-ErGO/GCE has an excellent detection performance for OTC, which was unmatched by the other electrodes. The result may be owed to the synergistic effect of ErGO and Ta_2_O_5_, further enhancing the current of the peak. Thus, in this work, Ta_2_O_5_-ErGO/GCE is the optimal choice for the determination of oxytetracycline.

### 3.4. Electrochemical Kinetics of Oxytetracycline on Ta_2_O_5_-ErGO/GCE

**Figure 5:**
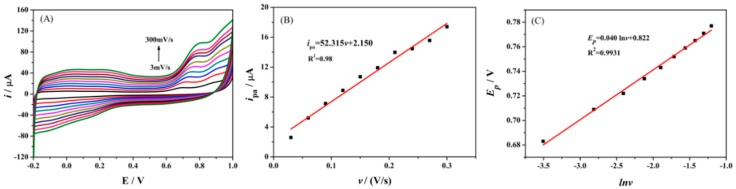
(**A**) Cyclic voltammograms of oxytetracycline on Ta_2_O_5_-ErGO/GCE at different scanning rates; (**B**) relationship between oxidation peak current (*i*_pa_) and scanning rate (v); (**C**) the oxidation potentials (*E*_p_) are linearly proportional to the Napierian logarithm of scan rate (ln*v*) (the current values have been baseline-corrected).

For a deep exploration of the reaction mechanism, the cyclic voltammograms of oxytetracycline (1.0 × 10^−5^ M) were plotted under different scan rates (30–300 mV/s) on Ta_2_O_5_-ErGO/GCE. The result is presented in Figure 5A. Consequently, with increasing scan rate, the *i*_pa_ of oxytetracycline rose as did the background current. Probably, high scanning rates are able to enhance the charging current of the double layer. Additionally, the linear relationship between the anodic peak currents of oxytetracycline (*i*_pa_) and the square root of the scan rate (*v*) is shown in Figure 5B. The linear equation of *i*_pa(OTC)_ (μA) = 52.315*v*^1/2^ (V/s) + 2.150 with a correlation coefficient (R^2^) of 0.98, identifying that the electrooxidation of oxytetracycline belongs to an adsorption-limited process. Furthermore, for an adsorption-controlled and totally irreversible electrode process, the relationship between the peak potential and the scanning rate is based on the Lavrion equation [41]:

(2) $$E_{p}=E^{0}+\;(\frac{RT}{\alpha nF})\;\ln\;(\frac{RTk^{0}}{\alpha nF})\;+\;(\frac{RT}{\alpha nF})\;\ln v$$

where *E*_p_ is defined as the peak potential, E^0^ is defined as the formal potential (V), T is defined as temperature (298.15 K), α is defined as the electron transfer coefficient, n is defined as the electron transfer number, k^0^ is defined as the rate constant, F is defined as the Ferrari constant (F = 96485 C/mol), R = 8.314 J/(K·mol), and *v* is defined as the scan rate (s). The relationship between the oxidation peak potentials (*E*_p_) and Napierian logarithm of scanning rates (ln*v*) is shown in Figure 5C. Obviously, the oxidation peaks directly moved toward a positive potential in different degrees at various scanning rates. The oxidation potentials (*E*_p_) are linearly proportional to the Napierian logarithm of the scan rate (ln*v*) with the linear equation: *E*_p_ = 0.040 ln*v* + 0.822 (R^2^ = 0.9931). According to Equation (2) and the linear equation (d*E*_p_/d*lnv* = RT/αnF = 0.040), αn can be calculated as 0.64. As for an irreversible process, α is suggested to be 0.5, so n can be estimated to be 1. Thus, one electron (e^−^) participated in the oxidation of oxytetracycline on Ta_2_O_5_-ErGO-GCE.

### 3.5. Optimization of Determination Parameters

#### 3.5.1. Effect of pH

**Figure 6:**
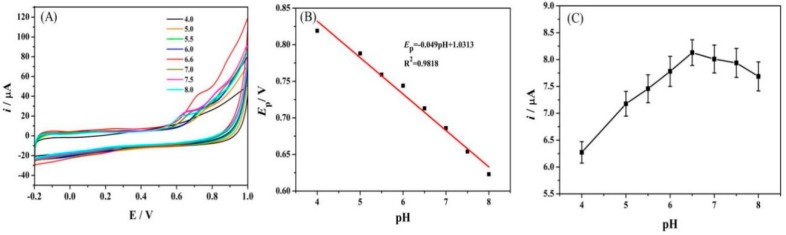
(**A**) CV pattern of 10 μM oxytetracycline on Ta_2_O_5_-ErGO/GCE at different pH; (**B**) the linear relationship of the anode peak potential of oxytetracycline and the pH; (**C**) the effect of pH on the current of oxytetracycline.

For the investigation of the effect of solution pH, the CVs of 10 µΜ oxytetracycline were recorded in different pH of PBS, as shown in Figure 6A. With the enhancement of pH, the oxidation peaks directly shift toward negative potential, and the peak potentials (*E*_p_) decrease linearly with pH, as depicted in Figure 5B. The linear equation of peak potential and pH is *E*_p_ = −0.049pH + 1.0313 (R^2^ = 0.9818), illustrating that protons (H^+^) participated in the electrochemical reaction of oxytetracycline. As Figure 6C depicts, with the increase in pH, the *i*_pa_ increases constantly until the pH is 6.5. Subsequently, the pH keeps aggrandizing, while the oxidation current of oxytetracycline decreases. Thus, pH = 6.5 is the optimal pH for detecting the oxytetracycline in this study.

#### 3.5.2. Effect of Deposition Parameters

**Figure 7:**
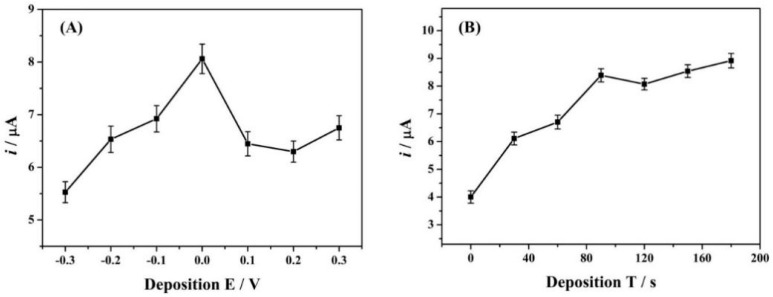
(**A**) Deposition potential and (**B**) deposition time on the oxidation peak currents of 10 μM oxytetracycline (0.1 M PBS, pH = 6.5) at Ta_2_O_5_-ErGO/GCE.

The influence of deposition potential for the current of the anodic oxytetracycline was investigated in the voltage range of −0.2–1.0 V. Oxytetracycline was deposited on the surface of Ta_2_O_5_-ErGO/GCE at numerous potentials for 90 s, and the *i*_pa_ of them were then carried out by CV. As Figure 7A shows, the peak current increases with the positive potential. When the deposition potential is over 0 V, the value of the peak current reduces, which is far less than that at 0 V. Thus, 0 V was suggested as the best deposition potential in this work. Furthermore, the effect of deposition time was studied at a fixed deposition potential of 0 V as well. In Figure 7B, it is evident to observe that the *i*_pa_ of oxytetracycline enlarges when the deposition time prolongs from 0 to 180 s. The *i*_pa_ gradually increases until 90 s first. However, after 90 s, the currents are unfluctuating and roughly the same as they are at 90 s. This result demonstrates that the deposition time can effectively improve the sensitivity of detecting oxytetracycline, recommending 90 s for the optimal choice. Therefore, the deposition was carried out at 0 V for 90 s in the following performances.

### 3.6. Selectivity Reproducibility and Stability Investigation

At the optimum analytical conditions, the selectivity performance was studied by measuring the response peak currents in the presence of potential interfering substances containing some metal ions and other antibiotics. The experimental results showed the 100-fold of Zn^2+^, Al^3+^, Ca^2+^, Mg^2+^, Cu^2+^, Na+, and K+, and the 20-fold of amoxicillin and tetracycline, which have no apparent influences on the response peak current of 10 μM oxytetracycline (signal change ≤ ±5%). The experimental results illustrate that the proposed Ta_2_O_5_-ErGO/GCE has high selectivity for the OTC determination.

For the evaluation of reproducibility, five different electrodes modified with Ta_2_O_5_-ErGO were measured in 10 μM oxytetracycline in 0.1 M PBS with a pH of 6.5. The relative standard deviation (RSD) for oxytetracycline was 2.34%, implying the good reproducibility. Additionally, the modified electrode stability was tested through running the cyclic voltammograms of 10 μM oxytetracycline, which was recorded approximately 25 times for 5 days. After the test, a drop of 13% was observed on the current signal of oxytetracycline. This shows that the Ta_2_O_5_-ErGO/GCE prepared have excellent stability.

### 3.7. Calibration Curve of Oxytetracycline

**Figure 8:**
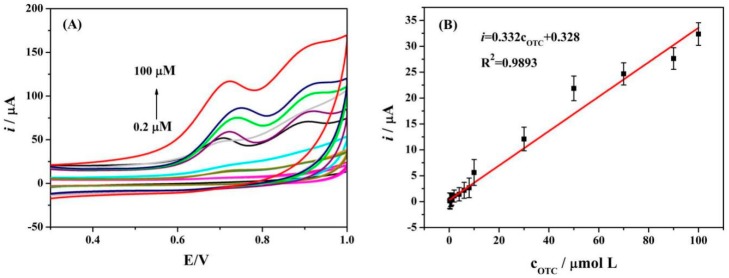
(**A**) Cyclic voltammetry obtained on Ta_2_O_5_-ErGO/GCE in 0.1 M PBS (pH 6.5) containing different concentrations of oxytetracycline (OTC) (0.2, 0.4, 0.6, 0.8,1, 2, 4, 6, 8, 10, 30, 50, 70, 90, and 100 µM). (**B**) The linear relationship between the anodic peak currents (*i*_pa_) of OTC and OTC concentration (c_OTC_) in the range of 0.2–100 µM.

The calibration curve for oxytetracycline at Ta_2_O_5_-ErGO/GCE was characterized by cyclic voltammetry under the optimal experimental conditions. The typical voltammograms are depicted in Figure 8A. In the range of 0.2–100 µM, the oxidation peak current is reasonably linear to oxytetracycline concentration (Figure 8B). The linear regression equation can be described as *i*_pa_ (µA) = 0.332c_OTC_ (µM) + 0.328 (R^2^ = 0.9893). According to the equations, LOD = 3S/N (S: Blank solution standard deviation; N: The slope of calibration plots), and the detection limit was identified as 0.095 μM. It mainly ascribes the excellent sensing performance to the synergistic effect of Ta_2_O_5_ and ErGO mentioned above.

In comparison to other electrochemical methods developed, as summarized in Table 1, this study provides a wide linear range and detection limit, which is relatively comparable to the fabricated electrodes reported for OTC determination. The minimum limit of detection indicates a good performance of the developed sensor. Thus, the modified electrode Ta_2_O_5_-ErGO/GCE can be used effectively for practical applications.

### 3.8. Detection of Oxytetracycline in Real Sample

CV was employed for quantitative analysis. The experimental results are listed in Table 2. There is no oxytetracycline in the milk supernatant sample. Besides, the good recoveries (100.1–120.9%) of the proposed Ta_2_O_5_-ErGO/GCE can be applied well to the OTC detection in real samples.

## 4. Conclusions

In this study, the Ta_2_O_5_ was synthesized and then composited with GO to obtain a Ta_2_O_5_-GO composition. Ta_2_O_5_-GO was dispersed in pure water and subsequently drop-coated on the surface of GCE. Subsequently, Ta_2_O_5_-ErGO/GCE was fabricated by the electrochemical reduction method. In comparing the bare GCE, GO/GCE, ErGO, and Ta_2_O_5_-ErGO/GCE, the oxidation signals of oxytetracycline were significantly enlarged on the nanocomposite-modified GCE (Ta_2_O_5_-ErGO/GCE). In addition, this modified electrode has successfully been used to eliminate the interferences of oxytetracycline. The linear range for the determination of oxytetracycline was 0.2–100 µM on Ta_2_O_5_-ErGO/GCE with the low detection limit of 0.095 μM (S/N = 3). The current developed method provides good sensitivity and selectivity for the detection of oxytetracycline in milk samples. Furthermore, this method has some evident advantages such as low cost, quick response, and simple fabrication.

## Figures and Tables

**Table 1:** Different modified electrodes for the detection of oxytetracycline.

Electrode	Electrochemical Techniques	Linear Range (µM)	Limit of Detection (µM)	References
Montmorillonite/acetylene black modified carbon paste microelectrode	^e^ DPV	0.5–50	0.087	[42]
^a^ Zn-Mt/GCE	DPV, ^f^ EIS	0.80–40	0.12	[43]
^b^ Pd-HSiO_1.5_/Ni–Co	Electrodeposition	400–0.01	10	[44]
^c^ CS-MWCNTs/ AuNPs	CV, amperometry	0.03–80	0.027	[45]
^d^ Ni-Co Alloy electrode	CV	10–500	10	[46]
Ta_2_O_5_-ErGO/GCE	CV	0.2–100	0.095	Current study

^a^ Zinc cation-exchanged montmorillonite-modified glassy carbon electrode; ^b^ hierarchical nanoporous Pd-HSiO_1.5_/Ni–Co composite electrode; ^c^ chitosan-multiwalled carbon nanotube composite multilayer films and gold nanoparticles; ^d^ nickel–cobalt alloy electrode; ^e^ differential pulse voltammetry; ^f^ electrochemical impedance spectroscopy.

**Table 2:** OTC in the milk supernatant sample by CV (*n* = 3).

Sample	Add (µM)	Total Found (µM)	RSD (%)	Recovery (%)
NO. 1	0.50	0.599	3.50	119.8
NO. 2	5.00	6.045	3.27	120.9
NO. 3	50.00	50.04	2.76	100.1
